# “You can’t put your luck on people”: a qualitative study of family views on the best interests decision-making process concerning adult kidney care in England

**DOI:** 10.1186/s12882-025-04147-7

**Published:** 2025-05-01

**Authors:** Jordan A. Parsons, Fergus J. Caskey, Jonathan Ives

**Affiliations:** 1 University of Birmingham, Birmingham, UK; 2 University of Bristol, Bristol, UK; 3 North Bristol NHS Trust, Bristol, UK

**Keywords:** Cognitive impairment, Decision making, Ethics, Mental capacity, Law

## Abstract

**Background:**

When an adult patient lacks decision-making capacity, care decisions must be made on their behalf in their “best interests”. We know little about the experiences of the family members of adult kidney patients with cognitive impairments, particularly in relation to best interests decisions. It is anticipated that they have varied experiences, with many feeling excluded from the most complex care decisions.

**Methods:**

This study aimed to understand the views and experiences of family members of adult kidney patients who had undergone a best interests decision in England. Semi-structured interviews (*n* = 6) were conducted with family members to explore their experiences and their views of the best interests process. Interview transcripts were then thematically analysed.

**Results:**

A range of experiences were reported, with four themes developed: prioritising patient preferences; family involvement; opposition to the best interests approach; and the importance of communication amongst all involved. Our findings suggest inconsistencies in how best interests decisions are approached in England, which can affect the nature and extent of family involvement. Participants highlighted the value of clear communication on all aspects of the decision-making process, including clarity on the roles of different stakeholders.

**Conclusions:**

When caring for adults who lack decision-making capacity, improvements in communication amongst all involved may minimise disagreements that escalate to legal proceedings.

## Introduction

Cognitive impairment in adult kidney patients presents challenges to making kidney care and treatment decisions [1]. There are many relevant examples of cognitive impairment, including long-term learning difficulties, deterioration through dementia, and temporary impairment through delirium. Studies have also demonstrated a high prevalence of cognitive impairment in kidney patients relative to the general population [2–4]. Contributors are not clearly defined but include age, cardiovascular risk factors, diet and exercise, and dialysis modality, amongst others.

Where a patient lacks the requisite capacity to decide about their care, it must be made on their behalf. In England, in the absence of a lawfully appointed proxy, such decisions are made by the patient’s doctor under the Mental Capacity Act 2005 (MCA 2005) in the patient’s “best interests”. However, the precise meaning of the term is elusive [5], as well as how much the patient’s own preferences are to be guiding. Those close to the patient – ordinarily family and friends, but sometimes also carers, faith leaders, and others well placed to speak to the patient’s views and preferences – are characterised as “consultees”. They hold a consultative role, helping the care team better understand the patient’s preferences.

Making decisions in line with the MCA 2005 can present many difficulties. There may be concerns over how to best respect the patient’s autonomy [6–7] and whether any previously recorded patient preferences remain accurate [8–9]. Then interactions between different parties may increase the difficulty. The literature suggests family members are more likely to favour life-sustaining treatment (meaning dialysis) [10–11], and we know that patients decline dialysis for various reasons [12–13], so ensuring those with cognitive impairments are not subject to dialysis against their best interests is vital.

The BIRD Study (Best Interests in Renal Dialysis) investigated how best interests decisions under the MCA 2005 are made in practice. Focused on decisions about dialysis and conservative kidney management (CKM), qualitative interviews were conducted with both healthcare professionals (HCPs) and family members with the aim of understanding their views and experiences of the decision-making process. This paper presents BIRD Study results from interviews with family members, which included six participants. Whilst a small sample, our data are the first to begin to explore the perspectives of this under researched and hard to recruit group and are thus an important contribution to understanding.

Whilst this study was in the context of England, the challenges of this area of decision making are internationally comparable [1]. Regardless of who is the *legal* decision maker when a patient lacks capacity, there will be others present whose input will have to be navigated. In particular, there is potential for confusion between the family’s own preferences, what the family are representing as the patient’s preferences, and what the patient’s actual preferences may be. There is, then, a pressing need to understand the perspectives of the different parties in these decisions.

## Materials and methods

### Design

This study aimed to understand the views and experiences of family members of adult kidney patients who had undergone a best interests decision in England. Qualitative methods were used to seek rich, detailed insights into the views and experiences of participants [14]. We conducted semi-structured interviews [15] using an evolving topic guide, prompting participants to explore areas of interest whilst affording them freedom to recount their views and experiences as they felt comfortable. The topic guide was informed by an earlier scoping review [1] and an example can be found in the supplementary material.

### Ethical approval

This study was reviewed by the Health Research Authority’s London – Camberwell St Giles Research Ethics Committee, with approvals granted on 22 December 2020 (REC reference: 20/LO/1233).

### Participants and recruitment

We recruited participants from two kidney units in England between June 2021 and April 2022. Recruitment was assisted by local collaborators, who were members of the healthcare team with an interest in the research. The two sites were chosen based on their serving populations of largely different ethnicities – one serving a largely White population, the other Asian. A third site, based in an area with a large Black population, fell through due to COVID-19 pressures. Our primary inclusion criterion was that participants are or have been involved in a best interests decision about dialysis and/or CKM as a family member.

Participants also had to be able and willing to consent to participation and communicate in English. The use of an interpreter was not feasible given study resource constraints.

We used purposive sampling to maximise diversity. That included various relationships to the patient; demographic range; and both positive and negative experiences (local collaborators were asked to actively seek participants who had negative experiences).

Potential participants were first identified and approached by a local collaborator based on recall and reviews of patient records. Those interested in participation were put in touch with JAP and provided with participant information materials. Eligibility was confirmed before scheduling the interview. Participants were offered the option of an in-person or online interview (via Zoom) due to COVID-19 measures. Online interviewing is not considered to significantly impact on the quality or quantity of data [16].

Informed consent was given before each interview, either written or oral depending on interview location. Oral consent was audio recorded and stored separately to the interview recording. After interview, participants were given a £20 shopping voucher as a token of appreciation.

### Data generation

All interviews were conducted by JAP. Participants were unknown to JAP, who introduced himself as a non-clinical PhD researcher.

Interviews were structured around a topic guide, amended periodically in response to interviews. Participants were informed they could take a break or terminate the interview at any point without providing a reason. A distress protocol was used, based on that of Draucker and colleagues [17].

### Data analysis

Reflexive thematic analysis was used, following Braun and Clarke’s stages of: data familiarisation, inductive coding, constructing themes, reviewing themes, and naming themes [18–19]. This is a suitably flexible approach to analysis that allows for in-depth exploration of individual experiences whilst interpreting connections that make for “themes” [20].

Audio recordings were transcribed verbatim by a transcription service [21] and checked for accuracy by JAP. All transcripts were read by JAP before any formal analysis to ensure familiarity, recognising the length of time between the first and last interviews. NVivo software (version 12) then facilitated inductive, data-driven coding by JAP. During coding, JAP maintained notes on candidate themes. Following coding, both codes and candidate themes were discussed between the authors before final themes were constructed and agreed. Regular discussions took place throughout the analysis period, allowing for any revisions to themes to be considered and agreed.

## Results

There were six participants – three at each site (see Table 1). The mean length of interviews was 1h04m, with a range of 0h51m to 1h20m. Four themes were developed from the data: prioritising patient preferences (see Table 2); family involvement (see Table 3); opposition to the best interests approach (see Table 4); and the importance of communication (see Table 5).

**Table 1:** Details of participants

Site	*n*=	Relationship to patient (*n*=)	Interview location (*n*=)
1	3	Parent (1) Spouse (2)	In person (3)
2	3	Child (3)	In person (1) Online (2)

### Prioritising patient preferences

Participants considered their role in decisions to be a proxy representative of the patient, providing answers they felt the patient would – and advocating for them (Q1). Several spoke of formalising this proxy role through lasting power of attorney for health and welfare (LPA-HW).

Whilst the patients concerned were deemed to lack capacity to decide about dialysis, participants reflected on attempts to involve the patient in decisions, recognition that the patient could still play some role (Q2). To that extent, participants took a supporting role in enabling the patient’s involvement.

For some, this entailed building protocols around the patient’s specific communication needs, particularly where the patient could not communicate verbally and those close to the patient had alternative means of communication. One participant explained a protocol devised with the patient’s school and provided to the care team (Q3). This protocol was designed to reassure the patient whilst allowing HCPs to draw on the relatives’ understanding of the patient’s unconventional indications of assent and dissent. This, reflected the participant, was “*a bit of education for the doctors*” (Participant 01).

There was nonetheless recognition of limits to the involvement of the patient given cognitive impairment, such that the patient could not decide even with additional supports. Some suggested efforts to involve the patient in these circumstances may be tokenistic, wherein the patient does not make – or is not anticipated to make – any meaningful contribution (Q4). A related concern was the patient’s disposition being to try to please HCPs, going along with things to avoid confrontation, which could limit the reliability of any preferences expressed (Q5).

Thus, there was greater focus on participants advocating for what they felt the patient would have wanted. This was sometimes informed by past conversations wherein the patient’s views were expressed, or reflection on the patient’s care decisions before losing capacity (Q6). Some participants reflected on the importance of the patient’s religious beliefs being adequately considered (Q7). One recalled conflict with the care team about the importance of keeping the patient alive in line with the patient’s own religious beliefs, finding it challenging when the care team does not seem to understand this (Q8). Whilst acknowledging that HCPs may not understand these views, there was frustration with not feeling heard when expressing them on the patient’s behalf.

Overall, participants viewed their role as advocate and source of information, providing an intimate knowledge of the patient that the care team lacks. They felt passionately about this, portraying a sense of duty to the patient in ensuring appropriate decisions. Many noted, however, that this was not always their role in practice.

**Table 2:** Quotes about prioritising patient preferences

Quote #	Quote
Q1	My role has always been- Well, you know, in the beginning, it was always as a daughter. It was always to think, “but what would my mum want? How is my mum going to feel about this?”. Especially after the stroke because she couldn’t speak for herself. It was always, yes, from a point of being a daughter and thinking that, you know, “mum would do this”, or, “mum would want that”. (Participant 05)
Q2	So, it wasn’t like my mum was ever left out or they didn’t acknowledge her. It never felt like that. They did come in, and as they got to know my mum, they knew what she was able to do and what she couldn’t maybe do. So, yes, but she was always consulted, and then we jumped in where we needed to. (Participant 05)
Q3	We encouraged them [the care team] to address the questions to us while keeping him in mind and reassuring- Holding his [P’s] hand, whatever. […] It was always a debate as to how much he could understand, even for us. We didn’t sign with him; we didn’t think he could cope with that. We used physical- We basically just hugged him and stroked him, and so forth, to reassure him, and encouraged everyone else to do the same. (Participant 01)
Q4	He was, but, yes, he was involved. We did discuss it with him. But that’s as far as, I suppose, it goes, really. […] So, in a sense, he probably wasn’t involved in that final decision about the haemodialysis. (Participant 02)
Q5	[H]e would have just said to the doctor, “do what you…”. He’s not a confrontational person at all. He would go along with whatever the doctor said he thought was best. […] I mean, it’s an acceptance because I think that’s the bit of the nature of dementia really, isn’t it, you know, sort of, “everyone else can make the decisions for me”. (Participant 02)
Q6	Okay, so at that point mum could talk. She could breathe, so she was involved in that decision. We had some dialogue with her, me and my sister, and it was very much a family decision once my mum was informed about what it means. She wants to live. She wants to stay alive. It was just a case of, “well, that’s what you need to do now, mum, if you want to stay alive”. (Participant 04)
Q7	[T]here are religious aspects that you’ve got to cover for people – individuals with religious beliefs of keeping someone alive, the treatment that they should get, the treatment that should be withdrawn from them. These kinds of things, if you live in the society that we’re living in, we have to take that kind of stuff on board. (Participant 06)
Q8	Some people [understood], but then you can’t blame someone not understanding it. They’re not ignorant – it’s just that they don’t know about it. (Participant 06)

### Family involvement

Participants spoke extensively of the nature and extent of their role in best interests decisions, especially regarding interactions with HCPs. Some felt they were actively making the decision rather than just contributing (Q9). Others felt more excluded by HCPs.

Several participants demonstrated clear understanding of their role in law, having arranged LPA-HW to ensure their decisional role (Q10) following past experiences of other relatives’ healthcare. Negative past experiences drove some to arrange LPA-HW – they were highly critical of the family role in best interests decisions. Those who felt somewhat sidelined felt it necessary to stand their ground (Q11). One suggested they were only permitted to care for the patient at home towards the end of their life because the family had previously provided that level of care to another relative. They felt the care team would not have allowed it “*if somebody had said what we were saying but hadn’t got the knowledge that my son had got [about caring for someone at the end of life]*” (Participant 03).

Whilst acknowledging there was often one relative acting as spokesperson to communicate with HCPs, participants spoke of wider family involvement outside the clinical setting (Q12). Sometimes, this caused internal family conflicts over what was in the patient’s best interests (Q13), partly owing to the flurry of emotions. The desire to ensure the “right” decision was made weighed on participants (Q14).

One reflected on the pressure of involvement in decisions and how some may prefer not to participate. Leaving the decision to someone else was suggested as a means of letting “*this faceless person take the blame for their own abilities or inabilities*” (Participant 04) should there be any negative consequences downstream. Others, however, found exclusion upsetting and wanted to hold someone accountable (Q15).

For most participants, the patient began kidney care as an adult. One, however, reflected on the patient being treated in a paediatric unit. They spoke of HCPs respecting what the parents did, making them feel nothing would be done without their knowledge or consent. When the patient transitioned to adult care, coinciding with a change in the legal role of parents, the participant commented on continuing to be heavily involved in decisions (Q16).

**Table 3:** Quotes about family involvement

Quote #	Quote
Q9	I always felt that the consultant and, as I said, the doctor on the ward, they were, normally, but on that one occasion, giving us the information and were definitely asking us to make the decision. Yes, so I never felt they were making the decision for us, no. (Participant 02)
Q10	I think you’d be asked questions, but if you didn’t have that power of attorney, then it would be that, in a way, you were being asked because it’s a polite thing to do to ask you what you think should be done. But the reality is if you don’t have power of attorney, whatever you’re trying to get done quite likely wouldn’t be done. (Participant 06)
Q11	Then we [members of P’s family] were able to come in together with our joint decision and say, “right. This is what we want and we are not budging”. Then they themselves [the care team] went, “oh, okay then. How do we go about making this happen?”. Then making it happen. […] Just because my mum can’t speak for herself it doesn’t mean that that need will not be met. (Participant 04)
Q12	[I]t’s a combined decision, let’s put it like that. But, to be fair, it’s probably more the whole family, rather than just myself and my husband [their husband being the patient]. (Participant 02)
Q13	I would say he [interviewee’s brother] has been the main decision-maker and I’ve been consulted all the way, but sometimes, you know, we did disagree on things. […] We had to work together the best we could and think about my mum. She was number one. I don’t know how, but we managed and we actually are in a good place now. (Participant 05)
Q14	[I]t was scary. It was like you don’t want to make the wrong decision. You don’t want her to suffer more than what she’s doing already. […] It is pressure. It is daunting. (Participant 05)
Q15	I remember we went in, we asked what happened because, all of a sudden, it happened to my mum. She came in fit and well. Obviously, you’re upset. That’s why you have all these questions. They are going to be natural questions, how it happened. You’re going to look to blame someone. Deep inside, you’re going to look to blame someone. It’s a natural thing that happens. (Participant 06)
Q16	It went on as before. I can’t remember an instance when somebody said, “he’s 18, it’s not for you to decide”. There was none of that, no. (Participant 01)

### Opposition to the best interests approach

Many participants were critical of best interests decision making. As noted earlier, several held LPA-HW to avoid a best interests decision being made, their discomfort with the process stemming from past experience. The general feeling was that the patient’s relatives should have a far greater role than the best interests approach provides – some went as far as saying it should be a family decision. LPA-HW was discussed as securing the family’s role as the best interests approach introduces an element of luck in which doctor ends up making the decision (Q17).

There was frustration expressed that the ability to advocate for their loved one was stunted in the absence of LPA-HW (Q18) and that it was the only way for them to formalise the proxy role they perceived themselves having (Q19). Some were confident the patient would have wanted to die at home, but felt the care team would not have allowed that without LPA-HW. One expressed serious concern about what would have happened in the absence of LPA-HW (Q20).

The overall concern of participants was a lack of legal guarantee of decision-making power for relatives, leading to varying degrees of opposition to the best interests approach. With the life and wellbeing of a loved one potentially on the line, participants felt it was important that they have some authority in the decision so that they can fulfil their self-perceived role as proxy and advocate. This led one to voice frustration for those who may lack knowledge of options such as LPA-HW and may thus find themselves involved in a best interests decision (Q21).

**Table 4:** Quotes about opposition to the best interests approach

Quote #	Quote
Q17	It doesn’t work. It doesn’t work at all. It’s a flawed system. That’s my God honest truth. It’s a flawed system. You know when you go to a cashier, you have some that are nice, some that are not nice, some that are going to smile? You can’t put your luck on people, “I might get a good service today”, and wish you have a good doctor. (Participant 06)
Q18	I think I’d reached the stage where I thought, if you like, I need to feel as though I would be in charge if I had to, and that if I had to make a decision, rather than just saying, “this is what I want”, I would need to have a document. I would need to have power of attorney. […] I think that you wouldn’t have the option to make the decisions if you didn’t have power of attorney. (Participant 03)
Q19	When you make a decision as an LPA holder, you can’t make a decision on your feelings. I can’t make a decision on how I feel. […] It’s what she would do. […] Now, I have views different to my mum, but my view doesn’t matter when it’s concerning her. (Participant 06)
Q20	So, had we not had the LPA, my mum would have been off the ventilator, and she wouldn’t be here today. That decision was made against the hospital and had to be kept because we knew the legal aspect of it, and that’s the only reason that, God willing, my mum is here. (Participant 06)
Q21	I don’t think there’s enough information out there for people to go and protect their families because a lot of people don’t know about LPAs and stuff that and how it could help. (Participant 06)

### The importance of communication

Cutting across the themes described above is the theme of communication – viewed by participants as essential to the best interests process. Participants considered it important for HCPs to communicate well not only with them, but with the patient – making a point of not excluding the patient because they cannot consent to care (Q22).

Many highlighted positive experiences whereby HCPs communicated very well (Q23). Others reflected on the limitations of resources provided, noting how visual aids instead of traditional written resources could improve understanding both for the patient and any consultees. Finding provided resources difficult to understand, some sought something more intelligible online (Q24). This was because they felt a responsibility to help the patient understand, requiring them to also understand.

Several participants recounted poor experiences, largely entailing HCPs being rather brusque in manner, skirting around the more interpersonal aspects of interaction. Some were more extreme. One participant detailed a situation of the family feeling accused of making poor decisions for the patient and ignored. Through several interactions with HCPs, they felt “*the LPA pretty much was thrown out of the window*” (Participant 06) in favour of the care team’s view of the patient’s best interests. HCPs raised formal concern over the LPA-HW, which was suspended pending investigation. For this participant, the process proved draining and felt like a fight (Q25). Whilst this was the only example of the family-HCP relationship breaking down to this extent, a similar sense of disenfranchisement was reported by many participants, felt to stem from poor communication.

There was a sense that clinical pressures and practicalities sometimes created communication issues. One participant described a particular phlebotomist’s ability to take the patient’s bloods when others struggled. Despite failed attempts by other HCPs and the patient being clearly distressed, requests that this phlebotomist be called were rebuffed (Q26). This participant acknowledged that “*you can’t just have a personal phlebotomist to come in whenever you want*” (Participant 01) but still felt the situation was poorly dealt with.

Beyond the nature of communication, several participants expressed concerns over content. Some felt HCPs were not always as forthcoming with information as they would have liked (Q27). They speculated that this was to protect them from too much information “*because normal people wouldn’t want to hear it*” (Participant 03). It was the failure of the care team to recognise that some would want to hear it that this participant objected to – a criticism of a perceived default position of protecting relatives from information overload.

Another participant felt resource pressures on HCPs explained a lack of information being provided. They described several aspects of the patient’s care relatives were initially told were not possible, only to later discover there were options available – for example, a mitten to stop the patient grabbing tubes (Q28).

Amongst those with LPA-HW, a view was expressed that “*maybe they’re [the care team] a bit more careful about what they say to you and maybe they give you more information than somebody that didn’t have it [LPA-HW]*” (Participant 03). The suggestion being that relatives without LPA-HW may be given selected information by the care team, preventing them from building a full picture of available options.

In contrast, one participant felt the doctor was “*very clear and did give both sides of the coin*” (Participant 02). When a decision between dialysis modalities had to be made, this participant did not feel pushed into a particular option, finding both to have been explained in comparable depth. Though this followed what was perceived as an assumption that dialysis would happen and all that remained was to choose a modality.

Continuity of HCPs was also considered important to communication. One participant spoke of an unpleasant experience with a doctor who was filling in for the usual consultant, in which that doctor advised against dialysis because “*people with dementia don’t look after things properly and they get infected*” (Participant 02). There was a sense that continuity enabled a good relationship between the patient, consultee(s), and HCPs given it is long-term care in this setting (Q29).

**Table 5:** Quotes about the importance of communication

Quote #	Quote
Q22	[H]onestly, one of the biggest things that I’ve learnt during this process, and still to this moment with my mum, all the staff, is communication. I know it sounds so fickle, but it is the biggest thing, you know, the communication. It’s like if you don’t understand something, then say. Don’t just go home thinking, “oh, I needed to ask this and I didn’t”. And they are willing to help. Yes, they are willing to help. They want you to be clear on what they’re going to do. (Participant 05)
Q23	It was a meeting of them letting us know what was going on, and then we’re giving our points. You know, it was very balanced. It was very equal. We were allowed to give our thoughts, views on my mum’s care. We were allowed to ask questions openly, and by this time, we had known them a while as well. So, it was quite comfortable, and we came out of there. Well, I came out of there knowing what’s going to happen, you know, what they’re doing with my mum, why they’re doing it and why they’re suggesting what they want to do. So, they were good. Those meetings, they were helpful. So, yes, that’s what I feel about them. (Participant 05)
Q24	So, we spent a lot of time before anything happened then, looking at the videos particularly, because they are animated and that does help a little bit. (Participant 02)
Q25	We were in a battlefield – we were fighting the world. I wasn’t taking care of my mum. I was fighting the world. I was fighting [hospital]. Me and my siblings were fighting [hospital]. (Participant 06)
Q26	Oh well, “no, I’m afraid he’s not on the ward or not available to answer”. You know, “in hospital you can’t always have who you want”. There was one occasion when two people had a go and I said, “this doesn’t work”. It was somebody quite senior and, I felt, rather fancied themselves at doing this slightly menial work. “Oh, I can remember”. Then failing. [Patient] would be in tears and it was very upsetting. (Participant 01)
Q27	I get the feeling that they only give you as much information as they think you need, not everything. They hold back a bit. (Participant 03)
Q28	There was a lot of misinformation. There was a lot of pressure, withholding truths. We found out later on that actually that’s not true. […] [T]he pressure is on them to clear the bed. (Participant 04)
Q29	It has been much better because we’ve not had to keep explaining things from the beginning. You know, we’ve built quite a good rapport with them, and they’ve got to know my mum. (Participant 05)

## Discussion

Participants clearly felt that, despite cognitive impairment, it is paramount that the patient is involved, as much as possible, in any care decision. This was seemingly characterised as respecting the patient’s autonomy, ensuring the patient’s views and preferences are central. To that extent, participants align with the principles underpinning the MCA 2005 (s.4(6) MCA 2005). However, there is divergence from the MCA 2005 in the decisional strength participants would afford the decision they think the patient would have made. They seemingly endorsed a substituted judgement model, wherein the patient’s assumed decision is followed even if thought to be poor. This is perhaps attributable to the confidence participants had in their knowledge of the patient’s preferences; they voiced no concerns about accurately identifying these. This may be cause for concern given literature demonstrating inaccuracies in such prediction, particularly in relation to the patient’s discontinuity of self [22–23].

Given this, participants spoke of conflict arising where they felt HCPs did not adequately recognise the individual circumstances of the patient or the patient’s personal views and preferences (including religious convictions). However, whilst some spoke of conflict, others felt that they were making the decisions with full support of the HCPs – which, if accurate, is at odds with the process outlined by the MCA 2005 where those without LPA-HW are concerned (s.4(7) MCA 2005). This speaks to existing literature, which highlights families being given an influential role as a form of “defensive medicine” [10, 23–24].

These conflicts led some participants to oppose the best interests system. Considering the limits on decisional power afforded to the patient’s relatives, several expressed a preference for some manner of substituted judgement, with the family acting as decision maker even in the absence of LPA-HW. Interestingly, amongst those who did not criticise the best interests system, most reported feeling that they had made decisions. A question remains over whether they would have opposed best interests if they had experienced the exclusion others reported.

Overall, the importance of good communication for collaborative decision making was considered central. Even if they might remain unhappy with how decisions are made, all participants valued HCPs being honest and forthcoming with information, not leaving them in the dark about their loved one’s care. This was equally true of those who strongly opposed the best interests decision-making framework stipulated by the MCA 2005. We anticipate the same being true across jurisdictions, even if the *legal* decision maker varies.

Given these findings, it is recommended that further training and resources are developed to support best interests decision making in this context. Training ought to focus on how healthcare professionals can navigate conversations about these complex legal decisions, whilst resources may benefit all stakeholders – patients, consultees, and professionals – by providing clarity on legal requirements and offering an outline decision-making approach. In addition to being a significant and novel contribution to the kidney care literature, our findings also contribute to the wider literature on decision making where patients have cognitive impairments. With the challenges of best interests decision making being widely recognised – including both misunderstanding and inconsistency [25–26] – the insight into the process we provide may be informative in other contexts. As such, we suggest there would be value in exploring the applicability of our recommendations in other care settings.

### Limitations

Our sample comprises a small number of family members recruited from two kidney units, so is not representative. However, qualitative research is not intended to draw on large sample sizes for the purposes of generalisability. Given how underexplored they are in the literature, the perspectives of relatives are of great importance. Whilst we wanted to recruit more, COVID-19 introduced a range of recruitment challenges.

Our sample also only includes family members. There remains a need to understand the perspectives of consultees that are not related to patients – IMCAs (independent mental capacity advocates), faith leaders, carers etc.

## Conclusion

Our findings highlight potential issues in best interests decision making in the kidney care context. Poor communication risks undermining the relationship between HCPs and relatives, maintenance of which is especially important in chronic care settings where discussions will often continue for many years. Equally, if family members are, in some settings, taking a deciding role in best interests decisions, this is contrary to the MCA 2005 and risks the preferences of those family members having too strong an influence. As such, acknowledging the potential for confusion between the family’s own preferences, what the family are representing as the patient’s preferences, and what the patient’s actual preferences may be, is necessary for good best interests decision making. We note, however, that a position of healthy questioning is not the same as an automatic cynicism about a family’s motives.

Overall, participants emphasised a strong element of luck in the best interests process around who is in the patient’s care team. Things that are highly valued – such as involving the patient in the decision and communicating clearly and honestly – were felt to be inconsistent across individual HCPs, leading some to wholly oppose the best interests system. This suggests there may be a need for greater consistency in how best interests decisions are approached by HCPs in kidney care.

## Data Availability

The datasets used and/or analysed during the current study are not open access, but are available from the corresponding author on reasonable request.
